# Influence of HLA-DRB1 Alleles on the Variations of Antibody Response to Tuberculosis Serodiagnostic Antigens in Active Tuberculosis Patients

**DOI:** 10.1371/journal.pone.0165291

**Published:** 2016-10-27

**Authors:** Fangbin Zhou, Xindong Xu, Sijia Wu, Xiaobing Cui, Lin Fan, Weiqing Pan

**Affiliations:** 1 Institute for Infectious Diseases and Vaccine Development, Tongji University School of Medicine, Shanghai, China; 2 Clinic and Research Center of Tuberculosis, Shanghai Key Lab of Tuberculosis, Shanghai Pulmonary Hospital, Tongji University School of Medicine, Shanghai, China; 3 Department of Tropical Infectious Diseases, Second Military Medical University, Shanghai, China; National Institute of Infectious Diseases, JAPAN

## Abstract

Serology-based tests for tuberculosis (TB) diagnosis, though rapid, efficient and easily implemented, have so far shown unsatisfactory levels of sensitivity and specificity, probably due to variations of the antibody response in TB patients. The number and types of seropositive antigens vary from individual to individual. The person-to-person variations of antigen recognition may be linked to genetic polymorphisms of the human leukocyte antigen (HLA) class II alleles. In the present study, we find that there is a significant increase in the frequency of HLA-DRB1*14 (P = 2.5×10^−4^) among subjects with high antibody response levels compared to those with low antibody levels. HLA-DRB1*15, the most frequent allelic group in the studied active TB population, positively correlates with subjects with low antibody response levels rather than subjects with high antibody response levels (P = 0.005), which indicates the loss of relevant antigens for screening of patients with this allelic group. The potential association between HLA-DRB1 allelic group and individual antigens implies that TB diagnostic yield could be improved by the addition of antigens screened at the proteome scale in infected subjects from the HLA-DRB1*15 allelic group.

## Introduction

Despite substantial progress toward the goal of tuberculosis (TB) elimination, it continues to be one of the most prevalent infectious diseases worldwide, especially in developing countries. An estimated 8.6 million new cases and 1.3 million deaths are reported annually [1]. Efforts in recent decades to control this disease have met only limited success, gradually slowing the rate of increase but failing to eliminate TB. Moreover, the spread of HIV/AIDS in TB-endemic regions, and the global emergence of multidrug-resistant tuberculosis (MDR-TB) and extensively drug-resistant tuberculosis (XDR-TB), impede efforts to control and eliminate TB [2].

It is increasingly realized that the lack of rapid, effective and accurate diagnostic tools contributes to the high prevalence of TB worldwide. As a result, recent efforts involving high-throughput screening of the entire *Mycobacterium tuberculosis* proteome have been made aiming at the identification of protein biomarkers of *M*. *tuberculosis* infection and disease [3–5]. Some promising diagnostic antigens have been found. Of these, the 38-kDa antigen has been the most frequently studied and is a major component in some commercial immunodiagnostic kits [6, 7]. Unfortunately, it was tested with recognition frequencies ranging from only 20.6% to 52.5%, largely depending on the characteristics of the study population used [8–10]. LprG is an immunogenic lipoprotein in *M*. *tuberculosis* identified as a T cell antigen [11, 12]. It was recognized by approximately 7.9% - 44.1% of sera from active TB patients [3, 13]. Mpt64 was reported as under phase III clinical trials to evaluate its potential as a substitute for tuberculin purified protein derivative (PPD) in 2007 [14]. However, the sensitivity of this antigen varied from 11.9% to 65.5% [15–17]. Intriguingly, HspX and LpqH, latent infection-associated antigens [18–20], could also be used as a target for serology-based tests [21–25].

Thus, in the search for appropriate diagnostic antigens, no single TB antigen-based assay has so far achieved a satisfactory serodiagnostic performance due to the complexity of the human immune response to TB antigens, leading to the outcome that up to 30% of patients with active TB are seronegative [26, 27]. Another study from our laboratory identified a set of TB diagnostic protein markers including the five most frequently studied antigens (the 38-kDa antigen, LprG, Mpt64, HspX and LpqH) and three novel antigens (Rv1488, Rv1566c and Rv1623c) by a protein array technology[28] and showed that these antigens revealed highly variable antibody response. The number and types of seropositive reactive antigens varied greatly from person to person. We hypothesized that variations in specific antibody responses to TB antigens in different individuals may be linked to genetic polymorphisms of the human leukocyte antigen (HLA) class II alleles. In fact, there is some evidence that the HLA alleles play a crucial role in the modulation of the immune response and can influence the outcome of TB infection [29, 30]. Other infectious diseases, such as hepatitis B and malaria, are also found to show association between HLA class II genes and antibody response to relevant antigens [31–33]. HLA class II alleles, including HLA-DR, HLA-DP and HLA-DQ, can regulate antibody production [34]. Considering HLA-DR alleles, HLA-DRB1, with more than 1700 known alleles at the population level, is one of the most diverse; it plays a vital role in the antibody response, with specific alleles influencing the acquisition of antibodies to various pathogen antigens [35, 36]. Here, we evaluate the potential influence of HLA-DRB1 alleles on the variations of antibody response to TB serodiagnostic antigens in active TB patients.

## Material and Methods

### Subjects

This study was conducted with approval of the Internal Review Board, Tongji University School of Medicine, China. All participants received information on the aim and procedures of the study, and written informed consent was obtained from all subjects. Active TB patients were recruited from Shanghai Pulmonary Hospital in China during the period from September 2012 to October 2014. The criteria for patients enrolled in the study were as follows: all patients were diagnosed with newly-treated, active TB according to the fourth edition of Guidelines for treatment of TB [37]. The active TB case was defined as a patient with a positive sputum culture for the *M*. *tuberculosis* complex or a patient who has been diagnosed with active TB by a clinician and has been decided to have TB treatment according to clinical diagnostic criteria. Diagnostic criteria included bacteriological and pathological outcomes, abnormal radiological manifestation and clinical response to anti-TB treatment consistent with active TB. The negative control group included subjects with no history of TB and being negative by T-SPOT.TB or QFT-G. In addition, participants were excluded if: (i) they were taking any immunosuppressive medical treat; (ii) blood samples had hemolytic reaction; (iii) they were not of Han ethnicity. All individuals with HIV, hepatitis infections or autoimmune disorders were excluded and more details regarding age, gender and sputum smear were listed in Table 1.

**Table 1:** Characteristics of the studied active TB patient population.

Predictor	Seropositive<break />(N = 111)	Seronegative<break />(N = 50)	*P* value
Age (Mean, 95%)	46.85 (43.38, 50.32)	43.54 (37.59, 49.49)	0.314
Gender (Ratio, Female/Male)	0.39 (31/80)	0.39 (14/36)	0.993
Sputum smear (+/-)	0.26 (23/88)	0.22 (9/41)	0.691
Period of enrollment (m, yr)	September, 2012-October, 2014		

### Recombinant Antigen Preparation

The eight selected antigens were expressed in *E*. *coli* and then purified by Ni-NTA affinity chromatography. Specifically, the genes of selected antigens were amplified by PCR with the H37Rv strain genomic DNA as a template. The purified PCR products were cloned into the Pet-28a expression vector and recombinant antigens were expressed with a C-terminal His-tag. The recombinant antigens expression in *E*. *coli Rosetta* (DE3; Novagen, Germany) was induced with 1 mM isopropyl-β-D-thiogalactoside (IPTG) and analyzed by Western blotting. Purified recombinant proteins were examined by sodium dodecyl sulfate polyacrylamide gel electrophoresis (SDS-PAGE) and quantified using the Beyotime BCA protein quantitation kits. The purities of each protein were determined by Quality One software.

### Antibody Assays

IgG antibodies were detected by indirect ELISA against eight TB serodiagnostic antigens. Briefly, the purified recombinant antigens were diluted (final concentration 0.5–2 μg/ml) in coating buffer (0.05 M Na_2_CO_3_-NaHCO_3_, pH 9.6) and coated on 96-well Immunosorp plates (Nunc, Thermo Fisher Scientific Inc., Waltham, MA, USA) overnight at 4°C. Plates were washed thrice with 375mL of PBS-T (137 mM NaCl, 2.7 mM KCl, 10 mM Na2HPO4, 2 mM KH2PO4, and 0.05% Tween-20) and then blocked in 0.2 ml 5% skimmed milk for 2 h at room temperature. After three washes tested serum samples (diluted 1:50 in blocking buffer) were added and incubated at 37°C for 1 h. Plates were washed thrice and HRP-conjugated secondary antibody (1:10,000) was then added for 0.5 h at 37°C. Each plate well was washed five times before the color reaction was developed by TMB (3,3′,5,5′-tetramethylbenzidine) substrate solution and stopped by the addition of 2 M H_2_SO_4_. The optical density (OD) at 450 nm was measured using a microplate reader (ELX50, Bio-Tek Instruments, Winooski, VT, USA). A positive antibody test was defined as one with an OD value greater than the cutoff value, i.e., the mean OD value plus three SD from negative (healthy) control serum.

### HLA Typing

Human genomic DNA was extracted and obtained from blood clots by DNA tissue kit (Qiagen Inc., Chatsworth, CA, USA). DNA concentrations and quality were checked using a NanoDrop 2000 spectrophotometer (Thermo Fisher Scientific Inc., Waltham, MA, USA). HLA-DRB1 allele typing was performed using Sequencing-Based Typing (SBT) as described [38].

### Statistical Analysis

Heat map analysis was performed with R version 3.12 statistical software (The R Foundation for Statistical Computing, Vienna, Austria; available at http://www.r-project.org). Statistical analysis was performed using SPSS Version 20 software (SPSS Inc., Chicago, IL, USA). Frequencies of the HLA-DRB1 alleles were compared among various groups by the chi-square (χ^2^) test or Fisher's exact test, as appropriate, using standard contingency tables. The level of significance was set at P < 0.05, and an odds ratio (OR) with 95% confidence interval (CI) was calculated, when those comparisons showed significant P values, by using 2×2 contingency tables. Rare alleles, with fewer than five occurrences among all subjects, were categorized into a group called “other”.

## Results

### HLA-DRB1 Alleles in Active TB Patients

HLA-DRB1 typing and frequencies in active TB patients and healthy controls are summarized in Table 2. We found 13 HLA-DRB1* allelic groups in active TB patients. There were two predominant allelic groups in the cohort, HLA-DRB1*14 (13.98%) and HLA-DRB1*15 (18.32%). When compared with healthy controls, the group of active TB patients showed increased frequency of HLA-DRB1*04 (OR = 4.794 (1.434, 16.023), P = 0.005) and HLA-DRB1*15 (OR = 3.614 (1.742, 7.501), P = 2.7×10^−4^), and decreased frequency of HLA-DRB1*13 (OR = 0.229 (0.135, 0.387), P = 1.0×10^−5^).

**Table 2:** Frequencies of HLA-DRB1 alleles in active TB patients and healthy controls.

DRB1*	TB (n = 161)		HC (n = 77)		OR (95% CI)	χ^2^	*P* value
	N	%	N	%			
DRB1*03	44	13.66	25	16.23	0.817 (0.479, 1.392)	0.555	0.456
DRB1*04	28	8.7	3	1.95	4.794 (1.434, 16.023)	7.79	0.005**
DRB1*08	35	10.87	9	5.84	1.965 (0.920, 4.198)	3.316	0.077
DRB1*12	35	10.87	23	14.94	0.695 (0.395, 1.222)	1.609	0.205
DRB1*13	27	8.39	44	28.57	0.229 (0.135, 0.387)	33.449	1.0×10^−5^**
DRB1*14	45	13.98	25	16.23	0.838 (0.493. 1.427)	0.424	0.515
DRB1*15	59	18.32	9	5.84	3.614 (1.742, 7.501)	13.248	2.7×10^−4^**
DRB1*16	25	7.76	7	4.55	1.768 (0.747, 4.182)	1.721	0.19
Other^#^	24	7.45	9	5.84	1.298 (0.588, 2.863)	0.418	0.518

* P < 0.05;

** P < 0.01;

^#^ Other includes DRB1*01, DRB1*07, DRB1*09, DRB1*10, and DRB1*11.

### Antibody Responses to the Antigen Panel

**Fig 1:**
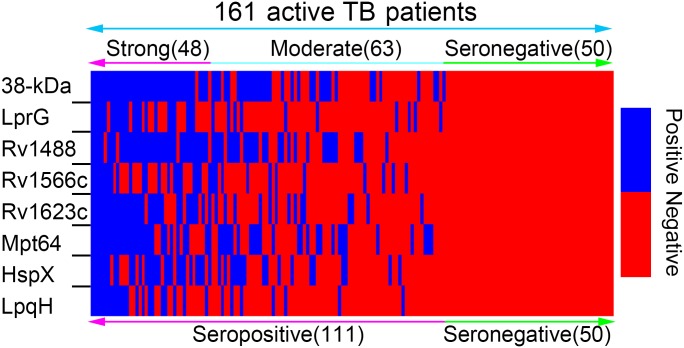
Antibody responses to the antigen cocktail. Antibody responses to eight serodiagnostic antigens in a panel of 161 archived infected serum samples are displayed in a heat map. The cohort was divided into two subgroups (seropositive and seronegative) according to antibody response level, and divided into three subgroups (strong response group (N≥4), moderate response group (4>N≥1), seronegative response group (N = 0)) according to the number of seropositive antigens recognized by different individuals, respectively. The optical density (OD) at 450 nm was measured using a microplate reader. As blue box indicated, a positive antibody test was defined as an OD value greater than the cutoff value, i.e., the mean OD value plus three SD from the negative healthy control serum. On the contrary, red box indicated a seronegative antibody test, which had an OD value less than the cutoff value.

Another study from our laboratory identified eight TB diagnostic proteins as shown in Table 3. In the present study, these antigens were employed to interrogate a total of 161 archived active TB patients (S1 Table). Fig 1 showed that up to 30% of patients with active TB were still seronegative. Moreover, antibody response in the cohort was variable. The number and types of seropositive reactive antigens fluctuated markedly from person to person. Some sera reacted with almost all TB antigens, while other sera reacted with none of the antigens. In most cases, the sera reacted with a variety of multiple antigens. As a result, the cohort was divided into two subgroups (seropositive and seronegative) according to antibody response level, and divided into three subgroups (strong response group (N≥4), moderate response group (4>N≥1), and seronegative response group (N = 0)), according to the numbers of seropositive antigens recognized by different individuals, respectively.

**Table 3:** Sensitivity of antigens associated with active tuberculosis in this study compared with literature values.

Antigen	Sensitivity (%)	Annotation
38 kDa	25.4 (ref 8)	Phosphate-binding lipoprotein PstS1
	20.6 (ref 9)	
	39.3 (ref 10)	
	52.5 (ref 17)	
	39.8 (this study)	
LprG	7.9 (ref 3)	Conserved lipoprotein LprG
	44.1 (ref 13)	
	18.6 (this study)	
Mpt64	11.9 (ref 8)	Immunogenic protein Mpt64
	65.5 (ref 15)	
	33.7 (ref 17)	
	31.7 (this study)	
HspX	19.9 (ref 15)	Heat shock protein HspX
	34.0 (ref 22)	
	62.0 (ref 23)	
	26.1 (this study)	
LpqH	39.0 (ref 8)	19 kDa lipoprotein antigen precursor
	82.0 (ref 24)	
	13.0 (this study)	
Rv1488	32.3 (this study)	Possible exported conserved protein
Rv1566c	18.0 (this study)	Possible Inv protein
Rv1623c	28.0 (this study)	Cytochrome D ubiquinol oxidase

### Frequencies of HLA-DRB1 Alleles in the Seropositive and Seronegative Subgroups

A total of 161 active TB patients were divided into seropositive and seronegative subgroups. The characteristics of the participants are described in Table 1. Age, gender and sputum smear were not significantly different between groups (P = 0.314, P = 0.993 and P = 0.691, respectively). The frequencies of HLA-DRB1 alleles in the two subgroups are shown in Table 4. Our results showed a significant increase in the frequency (18.02%, P = 2.5×10^−4^) of HLA-DBRB1*14 among the seropositive subgroup compared with the seronegative subgroup (3.00%). Moreover, we found a significant increase in the frequency (27.00%, P = 0.005) of HLA-DBRB1*15 among the seronegative subgroup compared with the seropositive subgroup (13.96%).

**Table 4:** Frequencies of HLA-DRB1 alleles in seropositive and seronegative subgroups.

DRB1*	Seropositive		Seronegative		OR (95% CI)	χ^2^	*P* value
	(n = 111)		(n = 50)				
	N	%	N	%			
DRB1*03	29	13.06	15	15.00	0.851 (0.434, 1.670)	0.219	0.64
DRB1*04	21	9.46	7	7.00	1.388 (0.570, 3.380)	0.525	0.469
DRB1*08	24	10.81	11	11.00	0.981 (0.460, 2.089)	0.003	0.96
DRB1*12	26	11.71	9	9.00	1.341 (0.604, 2.978)	0.523	0.469
DRB1*13	18	8.11	12	12.00	0.647 (0.299, 1.400)	1.236	0.266
DRB1*14	40	18.02	3	3.00	7.106 (2.143, 23.566)	13.439	2.5× 10^−4^**
DRB1*15	31	13.96	27	27.00	0.439 (0.245, 0.785)	7.934	0.005**
DRB1*16	16	7.21	9	9.00	0.785 (0.335, 1.843)	0.309	0.578
Other^#^	17	7.66	7	7.00	1.102 (0.442, 2.747)	0.043	0.835

* P < 0.05;

** P < 0.01;

^#^ Other includes DRB1*01, DRB1*07, DRB1*09, DRB1*10, and DRB1*11.

### Frequencies of HLA-DRB1 Alleles in the Strong, Moderate and Seronegative Response Subgroups

We next examined the polymorphisms of HLA-DRB1 alleles in 48 unrelated active TB patients with strong response, 63 with moderate response, and 50 with seronegative response (Table 5). There was a significant increase (15.63%, P = 0.002 and 19.84%, P = 1.4×10–4, respectively) in the frequency of HLA-DBRB1*14 among strong and moderate subgroup patients relative to the seronegative subgroup (3.00%). Moreover, we found a significant decrease (7.14%, P = 0.001 and P = 5.1×10^−5^) in the moderate subgroup in the frequency of HLA-DBRB1*15 compared with the strong subgroup and seronegative subgroup, respectively (22.92% and 27.00%).

**Table 5:** Frequencies of HLA-DRB1 alleles in the strong, moderate and seronegative response subgroups.

DRB1*	Strong group (n = 48)		Moderate group (n = 63)		Seronegative group (n = 50)		*P*1	*P*2	*P*3
	N	%	N	%	N	%			
DRB1*03	8	8.33	21	16.67	15	15.00	0.147	0.734	0.068
DRB1*04	11	11.46	10	7.94	7	7.00	0.28	0.791	0.374
DRB1*08	12	12.5	12	9.52	11	11.00	0.744	0.715	0.479
DRB1*12	10	10.42	16	12.7	9	9.00	0.738	0.379	0.6
DRB1*13	7	7.29	11	8.73	12	12.00	0.265	0.419	0.697
DRB1*14	15	15.63	25	19.84	3	3.00	0.002**	1.4× 10^−4^**	0.418
DRB1*15	22	22.92	9	7.14	27	27.00	0.509	5.1× 10^−5^**	0.001**
DRB1*16	6	6.25	10	7.94	9	9.00	0.469	0.775	0.63
Other^#^	5	5.21	12	9.52	7	7.00	0.601	0.497	0.231

* P < 0.05;

** P < 0.01; P1 = P value (strong vs. seronegative); P2 = P value (moderate vs. seronegative); P3 = P value (strong vs. moderate);

^#^ Other includes DRB1*01, DRB1*07, DRB1*09, DRB1*10, and DRB1*11.

### HLA-DRB1 Alleles and Antibody Response to Individual Antigens

Since we found that HLA-DRB1*15, the highest frequency allele (18.32%) in whole studied active TB population, positively correlated with subjects with low antibody response levels rather than subjects with high antibody response levels (P = 0.005), which implies the loss of relevant antigens for screening of patients with this allelic group, it was necessary to investigate the potential association between the HLA-DRB1 alleles and antibody response to individual antigens. As shown in Table 6, HLA-DRB1*04 may participate in an enhanced antibody response against LprG (P = 0.055). Similar observations were made for HLA-DRB1*03 with Rv1566c and HLA-DRB1*14 with Rv1623c (P = 0.003 and P = 0.008, respectively). Other antigens showed no association with particular HLA-DRB1 alleles.

**Table 6:** The association between HLA-DRB1 alleles and antibody response to individual antigens.

DRB1*	*P* value (38 kDa)	*P* value (LprG)	*P* value (Mpt64)	*P* value (HspX)	*P* value (LpqH)	*P* value (Rv1488)	*P* value (Rv1566c)	*P* value (Rv1623c)
DRB1*03	0.621	0.934	0.744	0.86	0.187	0.071	0.003**	0.055
RB1*04	0.725	0.055	0.183	0.299	0.838	0.686	0.314	0.716
RB1*08	0.975	0.484	0.422	0.115	0.764	0.072	0.43	0.376
RB1*12	0.259	0.81	0.132	0.115	0.764	0.072	0.746	0.755
DRB1*13	0.352	0.594	0.811	0.661	0.775	0.582	0.33	0.839
DRB1*14	0.293	0.874	0.343	0.316	0.309	0.614	0.226	0.008**
RB1*15	0.47	0.71	0.405	0.842	0.066	0.527	0.374	0.424
DRB1*16	0.09	0.855	0.681	0.232	0.436	0.356	0.417	0.647
Other^#^	0.504	0.422	0.235	0.542	0.18	0.089	0.2	0.89

* P < 0.05;

** P < 0.01;

^#^ Other includes DRB1*01, DRB1*07, DRB1*09, DRB1*10, and DRB1*11.

## Discussion

TB is the second leading cause of death from infectious disease worldwide. The challenges posed by *M*. *tuberculosis* infection require a renewed focus on therapy regimens, vaccines and diagnostics [39]. The demand for TB diagnostic biomarkers arises partly from the lack of suitable methods to detect *M*. *tuberculosis* or its products from host samples. Serological tests, though simple, cheap and feasible for point-of-care diagnostics, are of limited use, mostly because of unsatisfactory diagnostic performance [26, 27]. Previous serological researches indicate a variable antibody response to TB antigens, which is consistent with our present results [8, 27, 40]. Of the serum samples of active TB patients, when reacted with different antigens, some reacted almost exclusively with discrete antigens, while the others reacted with almost all antigens. However, in most cases, sera reacted with a variety of multiple antigens (Fig 1).

Variable recognition of antigens by serum antibodies in active TB probably may derive from multiple factors. One is the characteristics of the infected host. There seems to be a tendency toward a strong antibody response in older people as they are at increased risk of frequently contacting TB. In this study, however, the age and gender distributions showed no difference between subgroups (P = 0.314 and P = 0.993, respectively). A second factor may be bacillary load in the sputum. Though some results showed that antibody responses somewhat correlated with bacterial burden [3, 41], this was not observed for any subgroup in our panel, partly because conclusive evidence on this question requires a larger cohort of sera from active TB patients (Table 3). A third factor is the genetic background of the infected people. Several studies have indicated that variations in specific antibody responses to TB antigens in different infected individuals are associated with HLA genes [29, 30]. Other infectious diseases, such as malaria, also show correlation between HLA class II genes and antibody response to relevant antigens [32, 33, 36]. Indeed, polymorphism of HLA-II alleles has the potential to regulate antibody responses via the ability to bind exogenous as well as endogenous peptides and thereby change the nature of T cell recognition [42].

In the present study, we showed that the active TB population was diverse, presenting 13 HLA-DRB1 allelic groups with the highest frequency of HLA-DRB1*14 and HLA-DRB1*15 and the lowest frequency of HLA-DRB1*13 compared with healthy controls, as was also reported by some previous studies [43, 44]. The overall allelic groups of the studied population were consistent with the general South China population[45] and thus could be further evaluated. We found that there is a significant increase in the frequency of HLA-DRB1*14 among subjects with high antibody response levels compared with those with low antibody response levels. The results indicated that patients with high antibody response levels may recognize additional epitopes from the eight serodiagnostic antigens, which could thus potentially influence the association findings. Moreover, our results revealed that HLA-DRB1*15, one of two higher frequency alleles (present in 18.32% of the studied active TB population), positively correlated with subjects with low antibody response levels rather than subjects with high antibody response levels (P = 0.005), which may indicate the loss of relevant antigens for screening of patients with this allelic group. Statistical analysis revealed a potential association between HLA-DRB1 allelic group and individual antigens. As shown in Table 6, the antigen LprG seemed to be more easily recognized by infected people with the HLA-DRB1*04 allele (P = 0.055). Similar findings applied to Rv1566c with HLA-DRB1*03 and Rv1623c with HLA-DRB1*14 (P = 0.003 and P = 0.008, respectively). Obviously, although recent efforts involving high-throughput screening of the entire *M*. *tuberculosis* proteome have identified many protein biomarkers of *M*. *tuberculosis* infection and disease, potential valuable diagnostic antigens may have been missed. In this study, the limit of using only a few antigens of *M*. *tuberculosis* may explain the failure to detect any specific antibody response in up to 30% of active TB patients. We suggest a screen of a panel of infected people with the HLA-DRB1*15 allelic group at the proteome scale, so that further potential diagnostic antigens can be identified. Thus, serological tests based on a cocktail of genetic associated antigens could be used for the improvement of TB diagnosis.

In conclusion, we have found the influence of the HLA-DRB1 alleles on the variations of antibody response to TB serodiagnostic antigens in active TB patients. Our findings suggest that HLA-DRB1*14 may participate in an enhanced antibody response, whereas HLA-DRB1*15 probably inhibits response. The potential association between HLA-DRB1 allelic group and individual antigens implies that TB diagnostic yield could be improved by the addition of antigens screened from infected subjects with the HLA-DRB1*15 allelic group at the proteome scale.

## Supporting Information

S1 Table Analysis of the results of eight antigens antibody response. (XLSX)

S2 Table The whole HLA-DRB1 allelic profile of study population. (XLSX)
